# Family-effects in the epigenomic response of red blood cells to a challenge test in the European sea bass (*Dicentrarchus labrax*, L.)

**DOI:** 10.1186/s12864-021-07420-9

**Published:** 2021-02-09

**Authors:** Madoka Vera Krick, Erick Desmarais, Athanasios Samaras, Elise Guéret, Arkadios Dimitroglou, Michalis Pavlidis, Costas Tsigenopoulos, Bruno Guinand

**Affiliations:** 1 UMR UM CNRS IRD EPHE ISEM- Institut des Sciences de l’Evolution de Montpellier, Montpellier, France; 2 Department of Biology, University of Crete, 70013 Heraklion, Greece; 3 Univ. Montpellier, CNRS, INSERM, Montpellier, France; 4 Montpellier GenomiX, France Génomique, Montpellier, France; 5 Nireus Aquaculture S.A. Purgoulaki Kastella, 344 00 Evvoia, Greece; 6 Hellenic Centre for Marine Research (HCMR), Institute of Marine Biology, Biotechnology and Aquaculture (IMBBC), 715 00 Heraklion, Greece

## Abstract

**Abstract:**

**Background:**

In fish, minimally invasive blood sampling is widely used to monitor physiological stress with blood plasma biomarkers. As fish blood cells are nucleated, they might be a source a potential new markers derived from ‘omics technologies. We modified the epiGBS (epiGenotyping By Sequencing) technique to explore changes in genome-wide cytosine methylation in the red blood cells (RBCs) of challenged European sea bass (*Dicentrarchus labrax*), a species widely studied in both natural and farmed environments.

**Results:**

We retrieved 501,108,033 sequencing reads after trimming, with a mean mapping efficiency of 73.0% (unique best hits). Minor changes in RBC methylome appeared to manifest after the challenge test and a family-effect was detected. Only fifty-seven differentially methylated cytosines (DMCs) close to 51 distinct genes distributed on 17 of 24 linkage groups (LGs) were detected between RBCs of pre- and post-challenge individuals. Thirty-seven of these genes were previously reported as differentially expressed in the brain of zebrafish, most of them involved in stress coping differences. While further investigation remains necessary, few DMC-related genes associated to the Brain Derived Neurotrophic Factor, a protein that favors stress adaptation and fear memory, appear relevant to integrate a centrally produced stress response in RBCs.

**Conclusion:**

Our modified epiGBS protocol was powerful to analyze patterns of cytosine methylation in RBCs of *D. labrax* and to evaluate the impact of a challenge using minimally invasive blood samples*.* This study is the first approximation to identify epigenetic biomarkers of exposure to stress in fish.

**Supplementary Information:**

The online version contains supplementary material available at 10.1186/s12864-021-07420-9.

## Background

Because samples are easy to obtain, poorly invasive, and can be stored in large collections that may reflect variation in many parameters at both the individual and the population levels, blood is certainly the most commonly used tissue to check for and to monitor the response of cells, organs, or whole organism to environmental perturbations, to assess health status of organisms, and to diagnose metabolic impairments and dysfunctions in vertebrates. As a tissue subjected to systematic hormonal fluctuations by a centrally produced stress response, blood is especially used to monitor stress indicators at the molecular, cellular or physiological levels in teleost [1, 2]. Plasma cortisol (the main glucocorticoid hormone) as a primary physiological stress indicator and few metabolites such as glucose and lactate as secondary physiological indicators are certainly the most commonly assessed biomarkers of stress in fish [1]. These plasma biomarkers combine interesting advantages for stress monitoring (e.g., cheap data generation, nonlethal). Nevertheless, because the response of fish to stressors requires the consideration of a complex regulatory network of non-linear actions that could not be fully integrated by few parameters, it has been proposed that new technologies should give rise to new biomarkers for fish biomonitoring, especially to improve welfare in the farmed environment [3]. Indeed, the last decade has seen the emergence of a number of technologies for quantifying the molecular responses of fish to stressors at a genome-wide scale, including transcriptomics, proteomics, and epigenomics (e.g. [4–11]). Omics studies traditionally target key organs for stress monitoring such as the brain, the kidney, or the liver, but tissue sampling is generally lethal.

Because fish blood cells are nucleated and, apart from blood plasma in which cortisol, glucose, lactate and other metabolites are measured, also mobilized as part of the stress response in fish [12, 13], it is appealing to investigate if components of their genomic machinery may respond to environmental stressors and broaden the panel for poorly invasive stress monitoring. To data, the use of red blood cells (RBCs) in ‘omics fish studies has received little attention [14–16], and a single study specifically investigated RBC epigenome in steelhead (*Onchorhynchus mykiss*) [17].

After salmonids, the European sea bass (*Dicentrarchus labrax*) is certainly the most investigated marine fish species in Europe using molecular tools. It has been extensively studied over the last three decades, in both natural and farmed populations (reviewed in [18]). This includes the sequencing of its genome [19] and an increasing number of epigenetic studies [20–27]. In this economically important fish (approx. 200,000 t produced worldwide in 2018 [28]), epigenetic studies covered research areas important to fish farming including, e.g., sex determination [19, 24], the dynamics of epigenetic marks in sperm [25], the effects of temperature [23], or the epigenetic impacts of the onset of domestication [26]. However, only one of these studies was carried out at the genome-wide scale [26], others focusing at modifications of epigenetic profiles for reduced gene sets. None of these studies explicitly targeted ‘stress’ (but see [22]), and stress monitoring in the European sea bass remains largely evaluated using blood plasma (or serum) parameters (e.g. [29–32]). Some authors proposed alternatives based on, e.g., gene expression, but, by traditionally targeting tissues such as liver, brain or kidneys, they are invasive and fish are sacrificed in most of the cases (e.g. [33]). How the RBC methylome analyzed in minimally invasive blood samples may capture components of the stress response is actually missing in sea bass.

In this study, we adapted the epiGenotyping By Sequencing (epiGBS) protocol originally proposed by Van Gurp et al. [34] to assess the genome-wide epigenomic variation in the RBCs of *D. labrax* submitted to periods of acute stress during a 3 month challenge test. EpiGBS targets variation in cytosine methylation – the covalent addition of a methyl group to cytosine nucleotides – that has long been accepted as an important epigenetic modification in many organisms [35, 36]. This modification integrates a second restriction enzyme and further multiplexing of individuals. Our aim was to explore the changes in the epigenomic landscape of sea bass RBCs in pre- and post-challenge fish to initiate and to motivate the use of differentially methylated cytosines (DMCs) as putative biomarkers of stress.

## Results

**Fig. 1:**
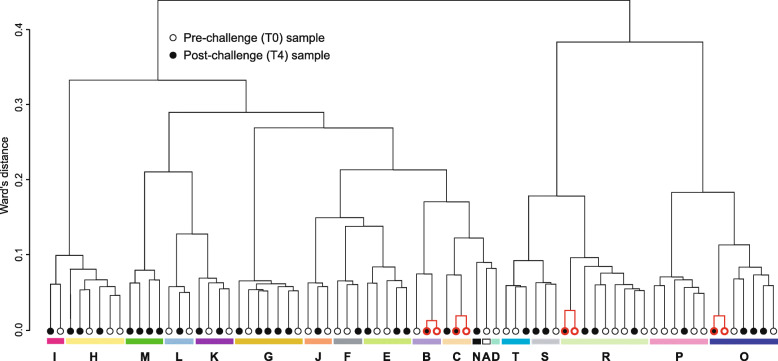
Hierarchical clustering based on of the 57 differentially methylated cytosines detected in this study. Capital letters refer to sea bass families and each family is associated to a single colour. Pre- and post-challenge samples (*N* = 74) are indicated. Samples highlighted in red correspond to the four individuals for which pre- and post-challenge blood samples were randomly caught. See text for details

Twenty sea bass families were produced to initiate a 3 month test in 6 month-old individual sea bass. This challenge was seeded with 20 individuals of each family (*N* = 400), minimizing tank effects. During the full challenge, fish were regularly submitted to acute stress, then could recover (see Methods section for details). In order to evaluate if this challenge could induce genome-wide methylation changes in sea bass RBC, a total of seventy-four randomly caught individuals (37 pre- [T0] and 37 post-challenge [T4] out of the 400 fish) were considered in this study. All individuals were submitted to the challenge, no unstressed individuals were available (see Methods section). While developed on a family-based experimental design, we only compared methylation difference between pre- and post-challenge juvenile sea bass and did not compare families in this study. Indeed, random sampling induced uneven representation of families within and among samples, and only nineteen out of 20 sea bass families were represented by at least one individual among the 74 samples analyzed in this study. Fish number per family ranged from one (families A, D, N) to nine (family R) individuals. Except for the families with a single representative and family M with post-challenge fish only (four), both pre- and post-challenge individuals were present in the 15 remaining families. Also because of random sampling, four individuals from four distinct families were retained twice by chance (Fig. 1). They were thus analyzed for both pre- and post-challenge conditions. A total of 70 distinct fish has been analyzed in this study.

### EpiGBS library construction and sequencing

We obtained 504,271,331 total sequencing reads of which 99.4% (501,108,033) were retrieved after trimming of our single library. After demultiplexing, read numbers per sample ranged from 2,284,915 to 16,314,759, with an average of 5,212,596 reads per sample (see Additional File 1). Demultiplexed samples were mapped against the *D. labrax* reference genome (~ 676 Mb) with a mean mapping efficiency of 74.5% (73.0% for unique best hits; Additional File 1). Sequencing reads mapped across all linkage groups (Additional File 2). The mean per base pair read depth was 250X.

### Methylation analysis

Out of the 10,368,945 CG dinucleotides present in the *MspI-SbfI* reduced-representation of *D. labrax* genome we obtained, 47,983 CpG coordinates were extracted with a minimum of 30X read depth and presence in at least 20 individuals. They were filtered out using a 15% methylation difference threshold and a nominal cut-off value of *q* < 0.001. With these parameters, only a total of 57 cytosines in CpG context were defined as DMCs between pre- and post-challenge sea bass (Table 1). Methylation differences ranged up to 46.4% for hypermethylated cytosines, and down to − 27.5% for hypomethylated cytosines. Hyper-methylation was more frequently detected than hypomethylation (11 [19.30%] hypo- vs 46 [80.70%] hypermethylated DMCs) in post-challenge sea bass. DMCs were distributed on 17 out of 24 LG groups and in or close to 51 distinct genes. Further information is provided in Additional File 3 (e.g. gene annotations, CpG context).

**Table 1:** Differentially methylated cytosines (DMCs) found in this study between pre- and post-challenge sea bass

*D. labrax* LG	Ensembl LG	*q*-value	methylation difference (%)	*D. labrax* Name	Gene ID	Gene Name	Location	References
LG1A	HG916837.1	0	-21,40	DLAgn_00094580	*FOXJ3*	forkhead box protein J3	*gb*	[37]
LG1A	HG916837.1	0	-21,67	–	*–*	–	*gb*	“
LG1A	HG916837.1	0	−15,51	–	*–*	–	*gb*	“
LG3	HG916843.1	1,54E-95	18,14	DLAgn_00141440	*ABLIM2*	actin binding LIM protein family -- member 2	*gb*	[37]
LG4	HG916844.1	1,08E-84	16,41	DLAgn_00145290	*CELA3b*	proproteinase E-like (elastase 2)		
LG4	HG916844.1	0	21,38	DLAgn_00146200	*NCBP2*	nuclear cap-binding protein subunit 2	*int.*	[37, 38]
LG5	HG916845.1	3,67E-265	21,91	DLAgn_00159160	*CILP1*	cartilage intermediate layer protein 1	*gb*	[38]
LG5	HG916845.1	8,93E-89	16,64	DLAgn_00159900	*GRPT1*	growth hormone regulated TBC protein 1	*gb*	
LG6	HG916846.1	6,44E-245	20,44	DLAgn_00164450	*GLG1a*	golgi glycoprotein 1	*gb*	[37, 39]
LG6	HG916846.1	0	−21,01	DLAgn_00166360	*GDPGP1*	gdp-d-glucose phosphorylase 1	*exon*	
LG6	HG916846.1	0	−18,98	DLAgn_00172370	*KBTBD13*	kelch repeat and btb domain-containing protein 13-like	*int.*	
LG6	HG916846.1	0	−18,18	–	*–*	–	*int.*	
LG6	HG916846.1	0	−16,12	–	*–*	–	*int.*	
LG8	HG916848.1	2,77E-247	22,52	DLAgn_00186250	*BTPF*	nucleosome-remodeling factor subunit bptf	*gb*	
LG10	HG916827.1	2,44E-79	15,44	DLAgn_00005890	*PBX1*	pre-B-cell leukemia homeobox 1	*gb*	[37]
LG10	HG916827.1	6,98E-125	21,55	DLAgn_00006210	*ADCY1*	adenylate cyclase 1 (brain)	*gb*	[37, 39]
LG11	HG916828.1	0	27,70	DLAgn_00016970	*MMNR2*	multimerin 2a Precursor Elastin Microfibril Interface Located Elastin Microfibril Interfacer	*gb*	
LG12	HG916829.1	0	22,32	DLAgn_00019700	*FBXO33*	F-box protein 33	*int.*	
LG12	HG916829.1	1,23E-128	20,01	DLAgn_00025280	*SASH1A*	Sam and sh3 domain-containing protein 1-like	*int.*	[37, 39]
LG14	HG916831.1	1,48E-53	15,74	DLAgn_00037440	*COL4A5*	Collagen type IV alpha 5 chain	*int.*	[37, 39]
LG14	HG916831.1	0	42,05	DLAgn_00037440	*COL4A5*	“	*gb*	“
LG14	HG916831.1	0	22,46	DLAgn_00038760	*ROBO3*	Roundabout homolog 2-like	*int.*	[37, 39]
LG14	HG916831.1	5,22E-212	17,93	DLAgn_00044110	*CLDN4*	Claudin 4	*gb*	[37]
LG14	HG916831.1	1,25E−155	27,48	DLAgn_00046110	*TMEM132E*	Transmembrane protein 132e	*gb*	
LG16	HG916833.1	2,78E-168	26,24	DLAgn_00063590	*CSMD3a*	CUB and Sushi multiple domains 3a	*gb*	[37]
LG16	HG916833.1	9,78E-92	17,12	DLAgn_00063770	*TRMT11*	tRNA methyltransferase 11 homolog	*int.*	[37]
LG16	HG916833.1	8,81E− 215	17,80	DLAgn_00064610	*FURIN*	Furin-like protease kpc-1	*gb*	[37, 38]
LG16	HG916833.1	0	17,76	DLAgn_00064820	*SPIRE1b*	Protein spire homolog 1-like	*gb*	[37]
LG17	HG916834.1	1,91E-82	18,84	DLAgn_00073160; DLAgn_00073170	*PLG* (sense)*; SLC22A2* (antisense)	Plasminogen (sense) / Solute carrier family 22 member 2-like (antisense)	*gb / 3’UTR*	[37]
LG17	HG916834.1	1,28E-231	19,86	DLAgn_00073770	*RRM2*	Ribonucleotide reductase regulatory subunit M2	*gb*	[37, 38]
LG20	HG916840.1	2,76E-207	15,01	DLAgn_00114460	*TNKSb*	Tankyrase, TRF1-interacting ankyrin-related ADP-ribose polymerase b	*gb*	[37, 39]
LG20	HG916840.1	1,34E-115	15,35	DLAgn_00114810	*LRRTM4L2*	Leucine-rich repeat transmembrane neuronal protein 4-like	*gb*	
LG20	HG916840.1	3,78E-68	15,32	DLAgn_00116160; DLAgn_00116150	*FICD* (sense)*; SART3* (antisense)	Adenosine monophosphate-protein transferase ficd-like (sense) / Spliceosome associated factor 3, U4/U6 recycling protein (antisense)	*gb*	[37]
LG20	HG916840.1	1,02E-88	18,04	DLAgn_00121270		Coding region of a truncated Non LTR Retrotransposable Element (RTE) RET-1_AFC	*rr*	
LG20	HG916840.1	0	35,61	DLAgn_00122640	*BMP3*	Bone morphogenetic protein 3	*gb*	[37]
LG20	HG916840.1	0	15,40	DLAgn_00124110	*ZMAT4*	Zinc finger matrin-type 4b	*gb*	[37]
LG22–25	HG916841.1	7,62E-96	16,89	DLAgn_00125750	*NOL4LB*	Nucleolar protein 4-like b	*gb*	[39]
LG22–25	HG916841.1	3,26E-99	15,05	DLAgn_00132500	*CCDC30*	Coiled-coil domain containing protein 30 like	*gb*	[37]
LG24	HG916842.1	2,81E-43	-15,63	DLAgn_00136190	*GLI2*	Zinc finger protein gli2-like	*gb*	[37, 38]
LG24	HG916842.1	2,87E-112	16,47	DLAgn_00136980	*LRRC3*	Leucine rich repeat containing 3	*int.*	
LG24	HG916842.1	0	30,68	DLAgn_00137190	*UNC80*	Protein unc-80 homolog isoform 2	*gb*	[37, 39]
LG24	HG916842.1	0	15,42	DLAgn_00137560	*CHN1*	N-chimerin	*gb*	[37]
LG24	HG916842.1	9,47E-204	17,08	DLAgn_00139980	*PTGFRN*	Prostaglandin f2 receptor negative regulator	*gb*	[37, 39]
LGx	HG916850.1	4,18E-251	19,94	DLAgn_00209310	*MYF5*	Myogenic factor 5	*gb*	
LGx	HG916850.1	4,52E-204	23,46	DLAgn_00209760	*CELF2*	Cugbp elav-like family member 2	*gb*	[37, 38]
SB-UN	HG916851.1	1,61E-152	16,29	DLAgn_00218300	*PRKCQ*	Protein kinase c theta type	*int.*	[37]
SB-UN	HG916851.1	1,09E-120	16,34	DLAgn_00220000	*PTPRB*	Protein tyrosine phosphatase receptor type B	*gb*	[37]
SB-UN	HG916851.1	6,07E-154	18,95	DLAgn_00222790	*NPAS3*	Neuronal PAS domain-containing protein 3-like	*gb*	[37]
SB-UN	HG916851.1	0	-21,55	DLAgn_00227120	*CRTC2*	CREB regulated transcription coactivator 2	*gb*	[37]
SB-UN	HG916851.1	6,11E-56	15,79	–	*–*	–	*gb*	“
SB-UN	HG916851.1	4,15E-61	17,53	DLAgn_00227130	*DENND4B*	Denn domain-containing protein 4b	*gb*	[37]
SB-UN	HG916851.1	1,51E-46	15,84	DLAgn_00236500	*GFRA2*	GDNF family receptor alpha-2	*gb*	[37]
SB-UN	HG916851.1	0	46,39	DLAgn_00238280	*DLG1*	Disks large homolog 1-like	*gb*	[38–40]
SB-UN	HG916851.1	9,43E-157	26,15	DLAgn_00242570	*BTR30*	E3 ubiquitin-protein ligase TRIM39-like (bloodthirsty-related gene family, member 30)	*gb*	
SB-UN	HG916851.1	6,99E-209	−17,71	–	*–*	–	*gb*	
SB-UN	HG916851.1	2,11E-216	−27,49	DLAgn_00244380	*GMPPB*	GDP-mannose pyrophosphorylase B	*exon*	[37]
SB-UN	HG916851.1	5,09E-256	20,97	DLAgn_00245980	*MATR3*	Matrin-3	*exon*	[37]

Location on the European sea bass linkage groups (LGs) of the 57 differentially methylated cytosines (DMCs) found in this study between pre- and post-challenge individuals. For each DMC, the false-discovery rate adjusted *q*-values at the nominal *q* = 0.001 cut-off threshold are reported, together with their methylation difference. Gene names and gene symbols (IDs) of DMC-related genes (*n* = 51) are reported. The location of each DMC is given (*gb*: gene body, *int*.: intergenic, 3’UTR or *rr*: repeat region). We did not arbitrarily defined promoter regions in this study. The right column indicates high-throughput stress-related neurotranscriptomic studies in which some of these DMC-related genes were reported as differentially expressed. It does not mean that these DMC related genes are involved *only* in brain-derived studies of stress (see Discussion for few reports). LGs are labelled as in [19] (GenBank assembly: GCA_000689215.1). An extended version of this table reporting annotations and further useful information are offered in Additional File 3

Most identified DMCs were located within identified gene bodies (44 out of 57, 77.19%), one in the 3’UTR regions of the Solute Carrier family 22 Member 2 (*SLC22A2*) gene on LG17, and one in a repeated region (a non LTR Retrotransposon Element on LG20). In the remaining cases (*n* = 11), DMCs are intergenic and located in a window ranging from 0.9 kb to 51 kb to the closest gene (respectively: *SASH1A* on LG12 and *TRMT11* on LG16; Table 1). Two pairs of overlapping, but inversely oriented genes share on their sense vs antisense strand an identical DMC: *PLG* and *SLC22A2* on LG17, and *SART3* and *FICD* on LG20 (Table 1).

When located on the same LG, DMCs were usually distant by at least 30 kb from each other. In only three instances, some DMCs were located close from each other (< 1.5 kb). These DMCs target the same gene (Table 1). This includes three hypo-methylated cytosines located ~ 1500 bp downstream of the predicted Kelch Repeat and BDB domain 13 (*KBTBD13*) gene with at most 88 bp between the cytosines. It also includes three hypomethylated cytosines (> 20%) on LG1A in the second intron of the forkhead box J3 (*FOXJ3*) gene. One other groups of two cytosines were found in the same exon (distant by 3 bp) of the *BTR30* gene (Table 1). A single DMC was associated to a repeat region and two DMCs were found to refer to the same gene (homologous to the *Gasterosteus aculeatus* paralogue of *COL4A5*, a collagen gene of type IV mostly implicated in the protein network of the basement membrane) (Table 1). For this gene, one DMC is located in the first intron while the second is 16.5 kb upstream of the start codon.

### Clustering

**Fig. 2:**
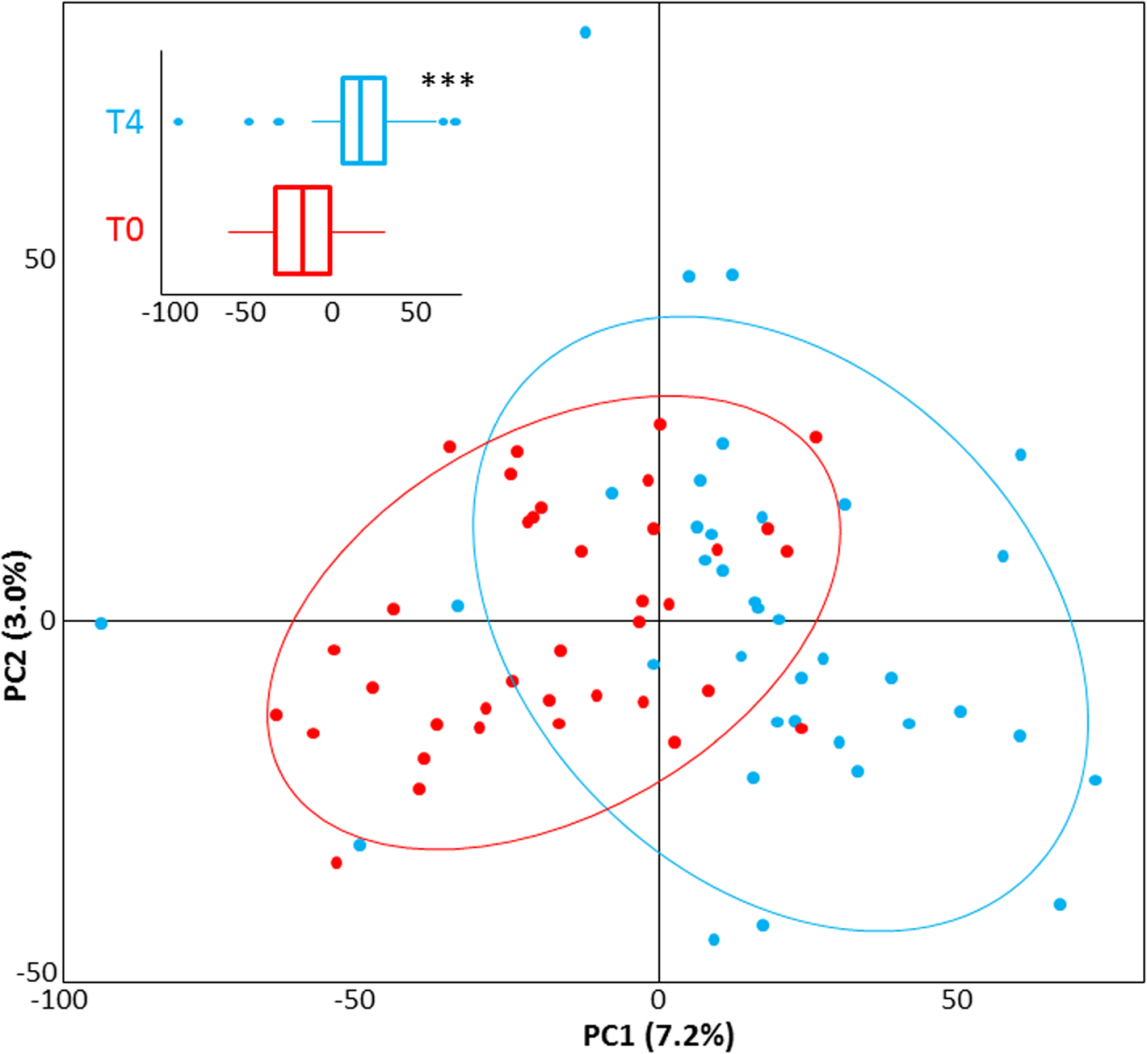
PCA based on the methylation profiles of the 57 differentially methylated cytosines (15% threshold) reported in this study (Table 1). Pre- and post-challenge individual sea bass (in red and blue, respectively) differ significantly along PC1 (*p* < 0.001), but not PC2. The insert illustrates the distribution of individual scores along PC1. Ellipses represent the 95% confidence limits over PC1 and PC2

Hierarchical clustering showed a strong family effect in methylation patterns (i.e. individuals within family clustered together; Fig. 1). The four individuals that were caught twice clustered together by pairs in all four cases. These individuals have the lowest levels of dissimilarity in hierarchical clustering, suggesting that family – not fully considered in our sampling scheme - may explain considerably more variation than treatment in their methylation profiles. Despite this strong family effect and clues of low impact of the challenge test on methylation, pre- and post-challenge groups can be distinguished based on their DMC profile in PCA. Mean loading scores of individuals were found significant among T0 and T4 for PC1 that explained 7.2% of total variance (Student *t*-test; *p* < 0.005, Fig. 2). No significant difference was found for loading scores along PC2 (3.0% of total variation; *P =* 0.404).

### Protein-protein interactions

Database mining screening for specific protein interactions on the String server revealed few possible pairs of associations between DMC-related genes (*n* = 8). These associations involve *ROBO3-CHN1*, *ROBO3-PRKCQ*, *ROBO3-LRRC3*, *DLG1-NCBP2*, *FURIN-PLG*, *PLG-MMRN2a*, *CELF-RRM2*, and *CRTC2-DENND4B* (see Additional File 4), with some them possibly linked to stress. *FURIN* - a subtilisin-like protein proconvertase – and plasminogen (*PLG*) processed proBDNF to mature BDNF (Brain-Derived Neurotrophic Factor), one of the most important molecule in fear memory (see Discussion). *ROBO3* and *CHN1* have been shown to interact with poorly-understood implications of *CHN1* in stress disorders [41]. *CELF2* (CUGBP Elav-like family member 2) and *RRM2* (Ribonucleotide reductase M2 polypeptide) are both known to participate to messenger RNA (mRNA) metabolism [42]. *CELF2* acts to post-transcriptionally stabilize mRNAs by relocating them to stress granules in the cytosol. *CELF2* interferes with *RRM2* that modulates its splicing activity. As post-transcriptional activities are at the core of methylation studies, the detection of this association seems relevant to our study.

## Discussion

We showed that a modified epiGBS protocol originally proposed by Van Gurp et al. [34] was applicable to further analyze patterns of cytosine methylation in RBCs of *D. labrax*. This is the first use of epiGBS in fish and the second in an animal species (Canadian lynx [43]). Overall, RBC’s DNA methylation was shown to respond to the challenge test, but observed changes were found mainly explained by the genetic background of individuals resulting from family-based effects, and involved relatively few sites and DMC-related genes.

### Mining the sea bass epigenome

The addition of a second restriction enzyme illustrates the flexibility of the epiGBS originally proposed by Van Gurp et al. [34] and more generally of reduced-representation bisulfite sequencing (RRBS) protocols for data acquisition and impact. The addition of a second restriction enzyme to a RRBS protocol in order to improve coverage and accuracy of CpG methylation profiling was however already shown [44], but hereby proposed in a context of improved multiplexing of samples.

The information provided in this study is based on the analysis of 47,983 distinct methylated sites distributed over all sea bass LGs. The mapping efficiency was high (74.5%) when compared to early values retrieved in human (~ 65%) [45], or in fish studies screening for genome-wide methylation (e.g. 55–60% in [46]; 40% in [17]). Other studies reported similar mapping efficiencies, but reported percentages of mapping for unique best hits that were generally lower. For example, in *Kryptolebias marmoratus*, Berbel-Filho et al. [47] reported a mean mapping efficiency of 74.2% but 61.1% unique best hits while, in this study, this latter percentage reached 73.0%. This reflects a more robust mapping of the DMCs we detected and significantly enlarge the breadth of the sites that can confidently exploit to retrieve functional information. Taking advantage of the epiGBS protocol that allow to process more samples [34], the number of individuals considered in this study is rather high (*n* = 70 distinct individuals), when most epigenomic studies in fish dealt with less than 30 individuals (range: *n* = 3 in [48]; *n* = 106 in [49] for a population study). In sea bass, Anastasiadi and Piferrer [26] previously reported a study that used 27 samples and as many libraries to be sequenced while our data were obtained from a unique library preparation. Our modified epiGBS protocol provides a considerable amount of information, certainly at a reasonable cost, to decipher methylation landscapes of sea bass or other species.

The operational and statistical thresholds used in the successive steps of this study are conservative, resulting in the discovery of a rather low total number of methylated sites, but certainly limiting the report of false positives. For example, a threshold of 30X and nominal cut-off value of 0.001 are quite conservative, when some studies might consider a threshold of 5X or 10X for a CpG to be analyzed and associated cut-off values of 0.05 or 0.01 (e.g. [26, 46, 50]). Relaxing thresholds would enable to retrieve more DMCs, but elevated thresholds should normally ensure that access to relevant information is reached. Thus, only 57 DMCs have been found in RBCs of pre- and post-challenge European sea bass. These DMCs were found mostly hypermethylated in post- compared to the pre-challenge individuals, and mostly located in gene bodies (i.e. the transcriptionally active portion of the genome) of fifty-one different genes. Differential methylation in gene bodies may regulate splicing and/or act as alternative promoters to reshape gene expression [51–53].

In addition to DMCs located in gene bodies, a dozen of DMCs were found in intergenic regions (21.0%). Intergenic cytosine methylation has been frequently described, including in response to stress [54], but its role remains poorly understood [55]. While numbers of genic vs intergenic DMCs may greatly vary, a ratio of ~ 80% of DMCs located in gene bodies and ~ 20% located in other genomic regions has been reported in other fish studies (e.g. [9]).

### An epigenomic perspective on stress biomarkers

Studies looking at the epigenomic landscape of RBCs in fish are scarce, and did not focus on response to a so-called stress challenge [17]. Our study yielded mixed results regarding this issue. Negatively, this study did not considered controls (i.e. unstressed fish) and it is difficult to assess if cytosines that were shown to respond to the challenge test really reflect the impact of stress or other parameters. This notably includes growth and ontogenetic changes in first year juvenile sea bass, together with sexual differentiation. Sexual differentiation occurs between 150 and 250 days post-fertilization in sea bass (e.g. [20]) and differential methylation measured in gonads at few candidate genes has been reported over this period [20, 23]. As our challenge test covers this period, results might be partially influenced by sexual differentiation. This has to be investigated further. However, we are not aware of studies that showed that differential methylation recorded in gonads might translate to RBCs, and none of the candidate differentially methylated genes previously studied in sea bass gonads was detected in this study. Methylation variation accompanying ontogeny and/or aging is reported in fish [56, 57], and it has been shown to be modified with age in sea bass muscles [27]. Unfortunately, methylation results also concerned candidate genes not detected as differentially methylated in this study. Relevant to our study, *BMP3* [58], *FURIN* [59], *NOL4B* [60], *Myf5* [61, 62], *NPAS3* [63], and *ROBO3* [64] are engaged in the development of the anterior region and/or the craniofacial skeleton which is known to be modified during sea bass farming [26]. Their roles were however studied in early development stages of mammals or zebrafish. As ‘epigenetic programming’ – apart of transgenerational inheritance - is mostly an early-life process that influence late-life effects (in fish, see, e.g., [5, 9, 38, 65]), we thus hypothesize that the epigenetic marks that could affect them would have been already present in 6 month-old sea bass that initiated the challenge test. Developmentally induced differential methylation acquired during the challenge test seems unlikely. Furthermore, some of them have clear relationships to stress exposure (e.g. *FURIN*, *NPAS3*; see below). However, in absence of dedicated study, we cannot totally rule out that methylation patterns observed in sea bass RBCs could also partly reflect the developmental or sexual regulation of a particular phenotype between pre- and post-challenge fish, rather than being directly related to the challenge.

Nevertheless - and more positively - some DMC-related genes detected in this study have been shown to be involved in the stress response in fish. Strikingly, 37 over 51 DMC related-genes were reported mainly from few neurotranscriptomics zebrafish studies that dealt either with reactive-proactive behavioural response to stress [37, 39] or with changes in social regulation that may promote stressful behaviour among congeners [40, 66] (Table 1). While not detected in RBCs but in brain tissues, correspondence across stress studies is interesting in this particular case. Indeed, while not investigated in fish so far, human stress studies have shown that blood cells responded to DNA methylation in the brain [67–69] (but see [70]), and specifically RBCs in birds [71]. Furthermore, several of these DMC-related genes are involved in the maturation of proBDNF to mature BDNF or the regulation of its activities (*ABLIM2, ADCY1b, CRTC2, FURIN, NPAS3, PLG,* and possibly *SLC22A2* and *DLG1*). BDNF consolidates both the within- and between-generation fear memory owing to epigenetic regulation [72, 73]. Its activity is strongly linked to glucocorticoid stress to imprint neurogenesis [74] and it acts as both a regulator and a target of stress hormone signaling [75]. BDNF is one of the target genes in fish stress studies (zebrafish [76], sea bream [77], sea bass [33, 78–80]), but its methylation status could not be investigated in this study as no *SbfI* restriction site is present within or close to this gene. However, the above-mentioned DMC-related genes might be linked to a ‘BDNF network’. The adenyl (ate) cyclase (AC, *ADCY1b* gene) is a brain-specific signaling enzyme that synthesizes the cyclic AMP [81]. This inducible signaling pathway participates to the synthesis of the active form of BDNF (proBDNF to mature BDNF) [82]. ProBDNF is processed by furin and the plasminogen system [83], including processing steps that necessitate actions of actin-binding LIM kinases (ABLIM) [84]. Stress imprinting at *FURIN* is likely and it has recently been shown that transgenerational epigenetic effects of furin activity were active in brain of mice [85]. Furin has also been shown to modulate learning abilities and memory [86]. ProBDNF cleavage by furin depends on brain AC and CREB (cAMP response element-binding protein) signaling [87, 88], but also plasminogen (*PLG*) [89]. This activity is modulated by stress hormones (corticosteroids) [90, 91]. One interesting supplementary observation is that CREB signaling necessary to furin is associated to *CRTC2* - a CREB co-activator. In mice, *CRTC2* is known to act as a switch for BDNF and glucocorticoids to direct the expression of corticotropin-releasing hormone (CRH) in the hypothalamus [92]. Additionally, in the brain, plasminogen encoded by *PLG* is converted to plasmin that cleaves BDNF in the extracellular synaptic domain [83, 93]. *PLG* has also been shown to regulate pro-opiomelanocortin (POMC) in the hypothalamic-pituitary axis, then the production of peptides hormones such as the adrenocorticotropic hormone (ACTH) [94].

Few other DMC-related genes should be mentioned. *SLC22A2* - also known as *OCT2* (organic cation transporter 2) – associated to the unique 3’UTR DMC found in this study is involved in numerous transmembrane transports [95], including at the blood-brain barrier [96]. It was found involved in memory in mice [97, 98] or *Drosophila* [99]. In this study, *PLG* and *SLC22A2* are associated to the same DMC; the functional significance of this situation needs further investigation. *NPAS3* (neuronal PAS domain containing protein 3) has a well-established action in memory [100, 101], and participate to a neural network that also includes BDNF [102]. *NPAS3* is also associated to the glial cell line-derived neurotrophic factor receptor-alpha2 gene (*GFRA2*) detected in this study and related to stress and anxiety [103]. Finally, *DLG1* (Disk-large homolog 1) plays a critical role in neural synapse formation, insulin secretion and glucose transport that are activated or modulated by stressors [104]. Adrenergic modulation implying *DLG1* was also found to correlate with emotional states and stress sensitivity in mice [105] and it indirectly participates to the regulation of BDNF as *DLG1* activates the glutamate receptor 1 (GluR1 [104];) that interacts with molecular processing of BDNF [106]. A relationship of *DLG1* with *NCBP2* (nuclear cap binding protein 2) was detected in protein-protein interaction analyses. *NCBP2* protect cellular RNA polymerase II transcripts from degradation and to guide them through the sequence of steps leading from transcription to translation [107]. As the cellular response to environmental challenges requires immediate and precise regulation of transcriptional programs, differences in cytosine methylation among pre- and post-challenge sea bass close the *NCBP2* gene could partly reflect the impact of the challenge.

While confounding factors may be present, results thus suggest that some DMCs reported in this study did not occur only by chance, are related to processes that regulate the hypothalamus-pituitary-interrenal (HPI) axis and hormones, but also to features that are expected to be developed or regulated during a challenge test (e.g. anxiety, fear memory, neurogenesis). While presumptive, these DMCs could effectively reflect the impact of the challenge test, and suggest that traditional blood plasma biomarkers could be potentially enriched by epigenetic marks to monitor welfare in cultured fish species. The link between brain and blood epigenomics remains however to be explored more deeply in fish and requires careful evaluation and validation to correct for tissue specificity, as requested in human [108]. Recent results on chicken RBCs are encouraging [71].

Hereby, we focused on possible relationships among DMC-related genes detected in this study and expressed in stress-related studies in brain tissues of fish [37, 39, 40, 66]. It should be however important to note that some DMC-related genes could be related to other components of the stress response (e.g., immune response, glucose metabolism). For example, a role of *PRKCQ* (PKC-theta, a protein kinase C theta type) in the immune response is well-known in vertebrates (e.g. [109]). *PRKCQ* is also known to participate to glucose metabolism, including glucose homeostasis [110]. One association with *DLG1* is reported, also related to the immune response [111]. The role of *CRTC2* on glucose homeostasis when facing stress has also been repeatedly reported [112–114], notably in relation to glucocorticoid levels [115]. We cannot expand further on this topic, but this suggests that DMC-related genes detected in this study may integrate several aspects of the stress response in fish.

### A family effect, but the possible absence of individual response

As in other fish species [116–118], a family effect imprinting the methylome was detected in this study. In parallel, results showed that the epigenetic profiles of the four individuals that were analyzed in the pre- and post-challenge conditions clustered very closely from each other. While based on few observations of randomly sampled individuals, the challenge had little impact on sea bass cytosine methylation landscape in comparison to family effects. Nature and strength of family-based epigenomic variation are of considerable importance attention to engage future selection breeding improvements in cultured fish like sea bass, including issues about health and welfare [119]. More generally, how transgenerational and within-generation stress-imprinting events may interact to shape both the plastic and the heritable component of the stress response in relation to environmental stimuli require in depth evaluation [120, 121]. To do so, far more complex and rigorous experimental designs that the one followed in the present study as to be adopted and temporal monitoring of the individual response of blood methylome to stress has to be promoted. In sea bass, such a research has been engaged for sex determination [24]. Results showed that some epigenetic marks were more likely engaged in transgenerational inheritance, while others be related to within-generation differences acquired during early development [24].

## Conclusion

Conclusions to this study are twofold. First, the European sea bass has become one of the most studied species in fish epigenetics [20–27], and for this species or for other cultured fish species, our modified version of the original epiGBS protocol seems to be a powerful and affordable method to screen a significant number of individuals with sufficient depth and coverage to reach meaningful conclusions. While this protocol should be compared to others (e.g. [44, 122, 123]), its use certainly deserves attention to design more integrated epigenomic-genomic studies [124, 125], as multi-omics investigations of stress, health and welfare [126, 127]. Second, as [17], we showed that RBCs are amenable to epigenomic investigations at a genome-wide scale in fish. RBC methylome revealed a family-based response to a challenge test. Efforts should be dedicated to validate some DMCs as stress biomarkers using improved experimental designs that would have, e.g., to set up baselines and to estimate context- and/or species-specific differences in order to enlarge the panel of diagnostic tools to monitor good practices in production setups [71].

## Methods

### Rearing, stress challenge, and blood sampling

Four hundred European sea bass were initially used in this study. Fish were produced in the hatchery facility of Nireus S.A. from breeders maintained in this company for scientific purposes. They resulted of a single crossing experiment (12 dams, 20 sires) that took place in January 2018. During the larval phase, 20 different families labelled from A to T were raised. Each family was reared separately in open circulation tanks at Nireus S.A. research facilities (Greece). To avoid environmental effects, fish were tagged at ~ 180 days post-hatch (July 10-13th 2018) and distributed in 20 tanks; each tank receiving 1 fish from each family (i.e., 20 fish per tank). Fish were fed twice a day, for 6 days a week, using a commercial diet (Blue Line 45:20 3.5 mm, Feedus S.A., Greece). Throughout the experimental period, the photoperiod was set at 12 L:12D, the water temperature and the salinity held constant (18.1 ± 0.2 °C and 28 ppt, respectively). Fish weights (mean ± SD) were 48.1 ± 12.8 g and 86.2 ± 24.1 g in pre- and post-challenge individuals, respectively.

Fish were submitted to one acute challenge test per month for three consecutive months, from July to October 2018. During this challenge, fish were exposed to high density stress by lowering water levels in the tank to 1/3 of the original volume, followed by chasing of the fish with a net for 5 min and a 30 min waiting period before sampling. These stressors are classical in sea bass studies regarding response to acute stress [29]. This protocol took place in each tank then for each fish individual entering the experiment. It was repeated for 3 consecutive times at 20–21 day intervals (period long enough for fish to recover).

An initial blood sampling occurred 2 weeks prior to the implementation of the stress challenge (hereafter T0, July 19th, 2018, pre-stress/control group) then at the end of the challenge test (hereafter T4, October 5th, 2018). Blood samplings were performed in anesthetized fish. Specifically, fish were anesthetized in 2-phenoxyethanol (300 ppm; Merck; 807,291; USA). Recovery was performed in a separate tank with provision of air before fish returned to their holding tank. Blood from the caudal vessel was collected. Plasma and RBCs were separated by centrifugation at 2000 g for 10 min. Careful separation of the plasma and RBCs was performed using 200 μl pipettes. RBC extracts were heparinized (heparin sodium; Sigma-Aldrich), transferred in microtubes and conserved at − 20 °C in 1 ml of RNA later. DNA was extracted using the Macherey Nagel Nucleo Spin Tissue DNA kit and quantified using a Qubit fluorometer (Qubit dsDNA BR Assay Kit, Q32853, Invitrogen). Thirty-seven blood samples at T0 (pre-stress) and thirty-seven additional samples at T4 (post-challenge) have been randomly selected for the downstream epigenomic analysis.

### Library preparation and sequencing

We followed the epiGBS protocol published by van Gurp et al. [34]. As the method was developed for plants (with methylation occurring in CpG, CHH, and CHG context; H being any nucleotide but a cytosine) and used a single digestion approach, the protocol was modified to make it more suitable and straightforward for our vertebrate system. Particularly, a double digest instead of the single digest approach was implemented. We chose the restriction enzyme *MspI*, a standard choice in reduced representation bisulfite sequencing-like (RRBS) studies, as its recognition site targets CpG rich regions [128]. A second enzyme (*SfbI*) with a recognition site length of 8 bp was used to reduce fragment numbers, and thus to increase read depth per fragment. The choice of this enzyme was guided by in silico digestion of the European sea bass genome [19] using *simRAD* [129]. This genome is available at: https://www.ensembl.org/Dicentrarchus_labrax/Info/Index.

One single library was prepared for a set of 74 samples. For this library, 200 ng of DNA of each sample were digested in a 40 μl reaction, using 0.25 μl *MspI* (NEB, 20,000 U/ml R0106S), 0.25 μl S*bfI*-HiFi (NEB 20,000 U/ml R3642L) and 4 μl of 10X cutsmart buffer. The reaction was run overnight at 37 °C. Unique forward and reverse adapter combinations allow multiplexing samples in the library. We added forward and reverse adapters in unique combinations (1 μl of adapter, 2.5 μM), 0.5 μl T4 Ligase (NEB, 400,000 U/ml m0202L), 6 μl of 10X T4 ligase buffer and 11.5 μl water were added directly to the digested DNA. Sequences of adapters are provided as Additional File 1. Adapters were ligated for 3 h at 23 °C followed by 10 min of enzyme inactivation at 65 °C. After ligation, all samples were pooled and one third of the total volume was used in the following step. The mixture volume was reduced using a Qiaquick PCR purification kit (28,104 Qiagen). The resulting product was cleaned a second time to ensure the removal of small fragments and adapter remnants using CleanPCR paramagnetic beads (Proteigene, CPCR-0050) with a ratio of 0.8X [sample:beads].

Since oligonucleotides were not phosphorylated (see Additional File 5), a nick translation was performed to repair the nick of the DNA at the restriction site and to fully methylate the hemi-methylated adapters. To do so, 19.25 μl of the concentrated and purified ligation pool were used in a 25 μl reaction, including 0.75 μl DNA Polymerase I (*E. coli*, 10,000 U/ml M0209S), 2.5 μl of 10 mM 5-Methylcytosine dNTP mix (Zymo Research, D1030), and incubated at 15 °C for 1 h. The library was then treated with sodium bisulfite using the EZ DNA Methylation Gold Kit (D5005, Zymo Research) following manufacturer instructions to convert unmethylated cytosines to uracil, paying attention to the optimal DNA amount per reaction. After bisulfite conversion, 14 cycles of PCR (95 °C for 1 min, 91 °C for 10s, 65 °C for 15 s, 72 °C for 10s and a final elongation step at 72 °C for 5 min) were performed, followed by a paramagnetic bead clean up with a 0.8X ratio. The library quality (fragment size distribution, no adapters left, no primers left; fragment size range from 300 to 800 bp; see Additional File 6) was verified on an Agilent 5300 Fragment Analyzer (Santa Clara, USA). It was sequenced (paired-end, 150 bp) on one lane of a SP flow cell on an Illumina™ NovaSeq 6000 at the MGX sequencing facility in Montpellier, France.

### Bioinformatics analysis

Raw sequencing data (NCBI accession number: GSE153838) were trimmed of low quality reads and adapter residues using *Trim Galore!* (v.0.6.4; available at https://www.bioinformatics.babraham.ac.uk/projects/trim_galore/). The trimmed reads were then demultiplexed using the process_radtags command of Stacks [130]. The option disable_rad_check was applied to avoid reads with bisulfite-modified restriction sites to be discarded. The demultiplexed reads were trimmed a second time on both 5′ and 3′ ends with the options --clip_R1 --clip_R2 and --three_prime_clip_R1 --three_prime_clip_R2 to remove introduced methylated cytosines during adapter ligation. Using the bismark_genome_preparation function of the program *bismark* [45], a bisulfite converted version of the European sea bass genome was prepared against which the demultiplexed and trimmed reads were mapped.

### Methylation analysis

The R package MethylKit [131] was used to determine differential methylation between pre- and post-challenge fish. CpGs with less than 30 read depth and with coverage > 99.9% of the distribution of read counts were filtered out to account for PCR bias. Coverage was normalized across samples, and only CpGs present in at least 20 out of 37 samples per group (56%) were kept for further analysis. Differentially methylated CpG sites were determined between pre- and post-challenge individuals using logistic regression (calculateDiffMeth function). Pre-stress individuals were considered as the baseline. Cytosines were considered as differentially methylated when presenting at least 15% methylation difference (as in [26] for epigenomic variation in *D. labrax*), and a nominal *q*-value < 0.001 between pre- and post-challenge fish. The combination of thresholds, sample size, and previously mentioned depth coverage ensures detection of biologically meaningful differences. Reads presenting DMCs were extracted using Geneious (v.11.0; available at https://www.geneious.com/) and mapped along LGs of the European sea bass reference genome to produce a primary annotation of potential candidate genes. As the annotation of the European sea bass and the sea bream (*Sparus aurata*) reference genomes have been recently released on Ensembl (http://www.ensembl.org/index.html), gene names and models have been controlled, eventually including a TBLASTN search in case of discrepancy.

### Data analysis

A principal component analysis (PCA) on the methylation profiles of individual samples at DMCs was performed to analyse the potential grouping structures within our data set. Differences in the distribution of mean individual loading scores for pre- and post-challenge fish were tested along each principal components axis using a Student *t*-test. Additionally, a hierarchical clustering was performed using Ward’s linkage method on Euclidean distances in order to explore whether sea bass families structure the data set.

We used String (https://string-db.org/) to investigate if DMC-related genes encoded for proteins are known to interact together. We used our gene list as input and the zebrafish genome as a reference for annotation. When one interaction was provided we specifically explored the literature for confirmation and relevant experimental evidence regarding stress.

## Supplementary Information

**Additional file 1.**

**Additional file 2.**

**Additional file 3.**

**Additional file 4.**

**Additional file 5.**

**Additional file 6.**

## Data Availability

Individual Illumina raw reads (one round of trimming) and processed files have been deposited in NCBI’s Gene Expression Omnibus [132]. Data set used in this study is accessible through Gene Expression Omnibus (GEO) Series accession number GSE153838 (https://www.ncbi.nlm.nih.gov/geo/query/acc.cgi?acc=GSE153838).

## Abbreviations

ABLIM: Actin-binding LIM kinase
AC: Adenyl(−ate) cyclase
ACTH: Adrenocorticotropic hormone
BDNF: Brain Derived Neurotrophic Factor
BMP3: Bone morphogenetic protein 3
BTR30: Bloodthirsty-related gene family member 30
cAMP: Cyclic adenosine monophosphate
CELF: CUGBP Elav-like Family Member 2
CHN1: Chimerin-1
CpG: Cytosine-phosphate-guanine
COL4A5: Collagen type IV alpha 5 chain
CRTC2: CREB regulated transcription coactivator 2
CREB: cAMP response element-binding
CRH: Corticotropin-releasing hormone
DENND4b: Denn domain-containing protein 4b
DLG1: Disk-large homolog 1
DMC: Differentially methylated cytosine
epiGBS: epiGenotyping By sequencing
FICD: FIC domain protein adenylyltransferase
HPI: Hypothalamus-Pituitary-Interrenal
FOXJ3: Forkhead box J3
KBTBD13: Kelch Repeat and BDB domain 13
LG: Linkage group
mRNA: Messenger RNA
LRRC3: Leucine rich repeat containing 3
MMRN2a: Multimerin 2a
Myf5: Myogenic factor 5
NCBI: National Center for Biotechnology Information
NCBP2: Nuclear Cap Binding Protein 2
NOL4B: Nucleolar protein 4B
NPAS3: Neuronal PAS domain protein 3
PCA: Principal component analysis
PC: Principal component
PLG: Plasminogen
POMC: Pro-opiomelanocortin
PRKCQ: Protein kinase C theta
RBC: Red blood cell
ROBO3: Roundabout guidance receptor 3
RRBS: Reduced representation bisulfite sequencing
RRM2: Ribonucleotide reductase regulatory Subunit M2
SART3: Spliceosome associated factor 3
SASH1A: SAM and SH3 domain-containing 1a
SLC22A2: Solute carrier family 22 member 2
TRMT11: TRNA methyltransferase 11 Homolog
UTR: Untranslated region
