# Supplementary material for: Family-effects in the epigenomic response of red blood cells to a challenge test in the European sea bass (Dicentrarchus labrax, L.)
Source: BMC Genomics. 2021 Feb 9;22:111. doi: 10.1186/s12864-021-07420-9 (PMC7871408; doi:10.1186/s12864-021-07420-9)

Additional File 2

Grapical representation of the coverage of the 24 linkage groups (LGs) of the sea bass genome by sequencing epiGBS reads.
Ordinates represent the mean number of sequencing reads. LG numbers are those used in [19] (GenBank assembly: GCA_000689215.1). Complex IDs resulting from the merging of previously defined LGs during final assembly (e.g. LG22-25 resulted in the fusion of previous LG22 and LG25). SB-UN (sea bass – unknown; hereby SB) indicates a composite LG made from unassembled scaffolds.

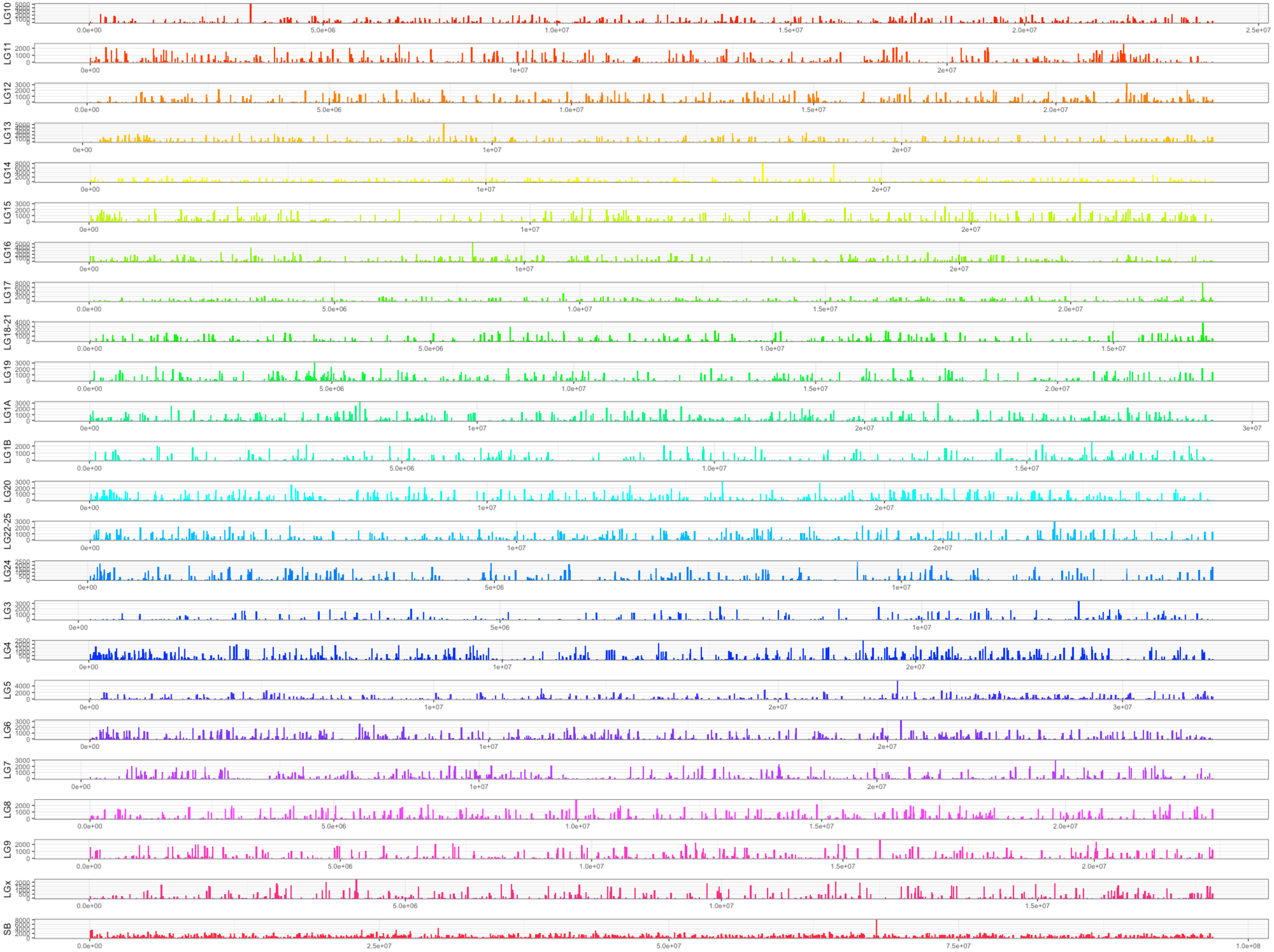

Supplement: Supplementary file 2 — Additional file 2. [file 12864_2021_7420_MOESM2_ESM.pdf]
