# Supplementary material for: Family-effects in the epigenomic response of red blood cells to a challenge test in the European sea bass (Dicentrarchus labrax, L.)
Source: BMC Genomics. 2021 Feb 9;22:111. doi: 10.1186/s12864-021-07420-9 (PMC7871408; doi:10.1186/s12864-021-07420-9)
Supplement: Supplementary file 4 — Additional file 4. [file 12864_2021_7420_MOESM4_ESM.docx]

**Additional File 4**

Output of the String search for protein-protein interactions using the list of sea bass DMC-related genes as input (Table 1, Main text).

Eight associations are shown. Note that the search was made using annotation on zebrafish *Danio rerio* and gene symbols might be different than those reported in tables. String automatically changes annotations to report only those used in the reference zebrafish genome. The correspondence is reported for the most significant (*CRTC2, NOL4LB, SASH1A*). *GLG1* and *CSMD3a* have no annotation in *D. rerio* genome and are not reported. Colored traits at each association have different meanings that are not relevant to (e.g. literature search, experimental evidence), but that have all been explored in this study. Gene names can be found in Table 1 or Additional File 3.
