# Supplementary material for: Family-effects in the epigenomic response of red blood cells to a challenge test in the European sea bass (Dicentrarchus labrax, L.)
Source: BMC Genomics. 2021 Feb 9;22:111. doi: 10.1186/s12864-021-07420-9 (PMC7871408; doi:10.1186/s12864-021-07420-9)

**Additional File 6**

Fragment size distribution of the sea bass epiGBS library sequenced in this study


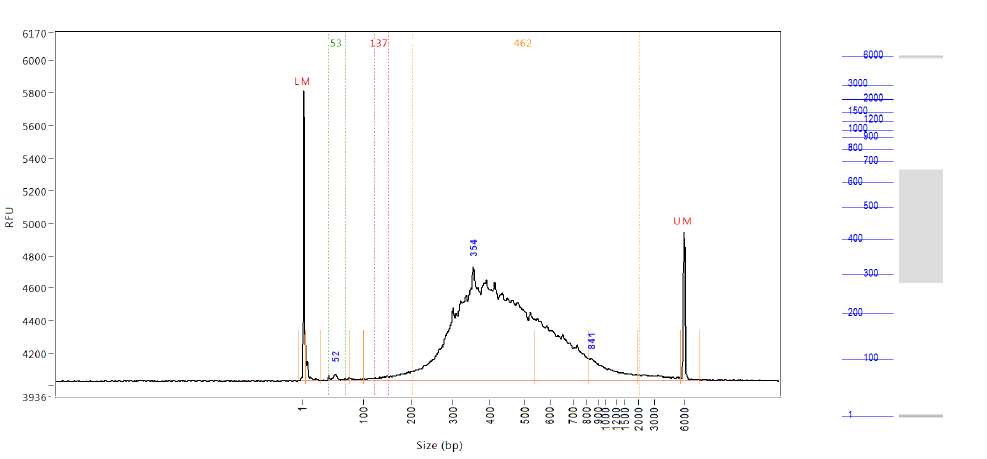

Supplement: Supplementary file 6 — Additional file 6. [file 12864_2021_7420_MOESM6_ESM.docx]
